# Cardiac biomarkers in pediatric CKD—a prospective follow-up study

**DOI:** 10.1007/s00467-022-05481-w

**Published:** 2022-03-16

**Authors:** Ylva Tranæus Lindblad, Georgios Vavilis, Milan Chromek, Abdul Rashid Quershi, Christian Löwbeer, Peter Bárány

**Affiliations:** 1 Divisions of Pediatrics, CLINTEC, Karolinska Institutet, Stockholm, Sweden; 2 Department of Pediatrics, Karolinska University Hospital, Stockholm, Sweden; 3 Huddinge BUMM, Paradistorget 4, 5tr, S-141 47 Huddinge, Sweden; 4 Department of Medicine, Karolinska Institutet, Stockholm, Sweden; 5 Division of Coronary and Valvular Heart Disease, Karolinska University Hospital, Stockholm, Sweden; 6 Renal Medicine, CLINTEC, Karolinska Institutet, Stockholm, Sweden; 7 Division of Clinical Chemistry, Department of Laboratory Medicine, Karolinska Institutet, Stockholm, Sweden; 8 Department of Clinical Chemistry at SYNLAB Medilab, Täby, Sweden

**Keywords:** Chronic kidney disease, Kidney transplantation, Troponin, NT-proBNP, Left ventricular hypertrophy, Left ventricular dysfunction

## Abstract

**Background:**

The N-terminal pro-B-type natriuretic peptide (NT-proBNP) and high-sensitive cardiac-specific troponin T (hs-cTnT) are associated with abnormal cardiac structure and function and an increased risk of cardiovascular death in chronic kidney disease (CKD) patients. There is limited knowledge about these cardiac markers in pediatric CKD patients.

**Methods:**

Longitudinal levels of NT-proBNP and hs-cTnT were analyzed in 48 pediatric patients, 22 with CKD (GFR range 8.8–68 mL/min/1.73 m^2^) and 26 transplanted patients (CKD-T; GFR range 30–99 mL/min/1.73 m^2^). Follow-up was scheduled after 1 and 3 years. Longitudinal patterns and associations to kidney function, cardiovascular risk markers, and echocardiographic parameters were assessed.

**Results:**

High NT-proBNP was present in 27% of CKD and 11% of CKD-T patients. Similarly 32% of CKD and 8% of CKD-T patients had elevated hs-cTnT levels. In longitudinal multivariate analyses, high log NT-proBNP was associated with low GFR (*β* =  − 0.01, *p* = 0.01) and elevated left ventricular mass index (LVMI; *β* = 0.02, *p* = 0.05). The strong association to LVMI remained when using GFR-adjusted NT-proBNP in similar analysis. Patients with left ventricular hypertrophy (LVH) also had higher NT-proBNP (235 [146–301] ng/L) than patients without LVH (86 [11–477] ng/L), *p* = 0.02. High hs-cTnT over-time was also associated with low GFR (*β* =  − 0.007, *p* = 0.01) and a low cc-TDI e´/a´, indicating a worse LV diastolic function (*β* =  − 0.09, *p* = 0.05). This association did not persist for GFR-adjusted hs-cTnT.

**Conclusions:**

NT-proBNP and hs-cTnT are elevated in pediatric CKD and CKD-T patients. GFR-adjusted NT-proBNP was associated with longitudinal levels of elevated LVMI suggesting this might be a marker for early subclinical myocardial damage.

**Graphical abstract:**

A higher resolution version of the Graphical abstract is available as Supplementary information. 
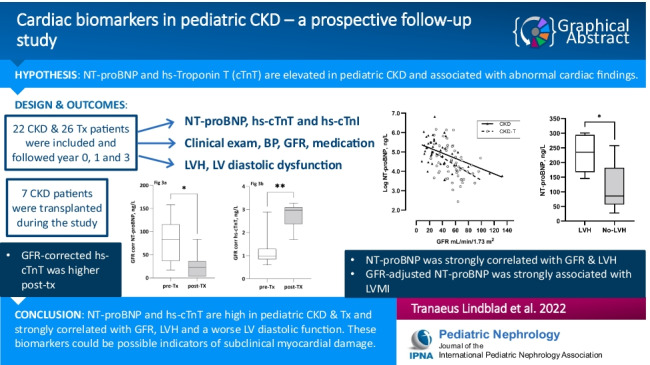

**Supplementary Information:**

The online version contains a graphical abstract available at 10.1007/s00467-022-05481-w.

## Introduction

Cardiovascular disease (CVD) represents one of the most important causes of death in chronic kidney disease (CKD) patients [1]. Several pathological mechanisms common in CKD increase the risk of developing CVD. Cardiac biomarkers are commonly used to assess various cardiovascular events, where cardiac-specific troponins (cTn) are characterized as markers of myocardial injury [2] and N-terminal pro-B-type natriuretic peptide (NT-proBNP) a marker of increased volume and cardiac load [3].

Troponins T and I (cTnT and cTnI) are widely used in adult cardiology to diagnose patients with acute myocardial infarction (AMI) [4], while NT-proBNP is used to assess patients with left ventricular hypertrophy (LVH), left ventricular dysfunction, and heart failure [3]. It is today established that both cTns and NT-proBNP are valid indicators of all-cause death and cardiovascular events in CKD patients and other populations [5, 6].

Importantly, patients with advanced CKD reveal elevated levels of these cardiac biomarkers, despite no obvious signs of AMI or heart failure. Two possible mechanisms are discussed, either continuous subclinical myocardial damage related to CKD comorbidities and/or reduced renal clearance per se [7, 8]. NT-proBNP is dominantly filtered directly by the kidneys, and the levels increase as GFR decreases [8] making it tricky to interpret in CKD patients. As most of the variation in cTnT also is explained by GFR [7], it is proposed that the levels of these cardiac biomarkers should be adjusted for GFR in CKD patients. The diagnostic value of cTnI is similar to cTnT, but compared with cTnT, cTnI has the advantage of being less influenced by kidney function [9].

Unfortunately, the risk of CVD and cardiac-related death is increased already in pediatric CKD [1]. While the risk decreases after kidney transplantation, it is still higher among pediatric kidney transplant recipients compared to healthy peers [10]. Indeed, the prevalence of preclinical cardiac changes like LVH and left ventricular (LV) diastolic dysfunction are high in these patients [11–13]. Large cross-sectional and prospective studies on adult CKD patients have shown that NT-proBNP and cTnT are associated with changes in left ventricular structure and function [14, 15]. Still, there are only few studies exploring the importance of cardiac biomarkers in pediatric CKD patients [16–19]. Analyzing cardiac biomarkers prospectively in children with CKD may improve the diagnostic accuracy and facilitate the prediction of CVD, thus improving clinical outcome.

The present study set out to analyze associations between longitudinal levels of high-sensitive (hs)-cTnT, hs-cTnI, and NT-proBNP and structural and functional cardiac abnormalities assessed by echocardiography in a cohort of pediatric CKD patients and kidney transplant recipients. Previously published algorithms to correct levels of NT-proBNP and hs-cTnT for kidney function based on adult studies were also assessed in this pediatric cohort, and in a sub-analysis, two different methods used to assess hs-cTnI were also compared.

## Subjects and methods

### Study population and design

The study was designed as an observational prospective cohort study of children with CKD, either non-dialysis CKD stage 2–5 patients (CKD) or kidney transplant recipients (CKD-T). All patients were treated at the outpatient Pediatric Nephrology Clinic at Astrid Lindgren Children’s Hospital, Karolinska University Hospital Huddinge in Sweden, with recruitment taking place between 2007 and 2008. The final study population consisted of 22 CKD and 26 CKD-T patients. The patients were seen at baseline, after 1 year and after 3 years. Seven CKD patients were transplanted during the follow-up period.

### Clinical characteristics

Medical records were reviewed for etiology of kidney disease, duration of CKD, time after kidney transplantation, and records of medication. Clinical data were collected including values for height, weight, body mass index (BMI), and office blood pressure.

Standard deviation scores (z-scores) for systolic and diastolic blood pressures as well as height, weight, and BMI were obtained [20, 21]. Hypertension was defined as systolic and/or diastolic blood pressure equal to or greater than the 95th percentile for age, sex, and height [20] and/or current treatment with antihypertensive medication. Obesity was defined as BMI equal to or greater than the 95th percentile for age and sex [21].

### Biochemical data

Blood was drawn in a standardized manner during a clinical visit in the morning and following an overnight fast. Serum analyses of hemoglobin, creatinine, cystatin C, calcium, phosphate, intact (i)-PTH, albumin, and high-sensitive (hs)-CRP were performed in all study participants. Early morning spot urine was also collected to assess albuminuria, with a cut-off set at urinary albumin ≥ 20 mg/L. GFR was assessed by using iohexol or inulin/PAH clearances in most assessments (92.4%). In the remaining patients (7.6%), GFR was estimated from cystatin C, or in a few patients where cystatin C values were not analyzed, GFR was assessed using creatinine levels [22].

The prevalence of anemia (hemoglobin < 5th percentile for age) [23], hypercalcemia, and hyperphosphatemia (albumin-adjusted calcium as well as phosphate level > 97.5th percentile for age) [24] were assessed. Secondary hyperparathyroidism was defined as i-PTH > 97.5th percentile corresponding to a level above 65 ng/L according to local laboratory standards.

Serum hs-cTnT and NT-proBNP were analyzed using electrochemiluminescence (ECL) immunoassays on the Cobas e 411 (Roche Diagnostics, Mannheim, Germany). Serum hs-cTnI was also analyzed and compared using two methods: STAT High Sensitive Troponin I immunoassay on the Architect Plus analyzer (Abbott Diagnostics, USA) and Access hs-cTnI on the DxI 800 system (Beckman Coulter, USA). Elevated levels were defined as > 97.5th percentile for age and sex for hs-cTnT and NT-proBNP [25, 26] and > 95th percentile for age for hs-cTnI (Architect) [27]. As there were no published reference values for children for Access hs-cTnI (DxI), we used adult reference values published by the manufacturer where the upper limit was defined as > 99th percentile for sex (11.6 ng/L for females and 19.8 ng/L for males) [28]. We used the female reference as upper limit for both sexes as the reference for Architect analysis only adjusted for age.

### Adjusting cardiac biomarkers for GFR

Due to the strong association to GFR for both NT-proBNP and hs-cTnT, we also assessed GFR-adjusted levels based on adult data [29, 30]. The following equations were used: GFR adjusted NT-proBNP = NT-proBNP/e^1,892 – 0.025 × GFR^ and GFR adjusted hs-cTnT = (GFR/90) × hs-cTnT. Depending on GFR, concentrations of the assessed GFR-adjusted hs-cTnT may be lower than the detection limit of the assay, 3 ng/L.

### Echocardiographic examination

Echocardiographic data were available for 19 CKD and 18 CKD-T patients (77%) at baseline, 18 CKD and 27 CKD-T patients (94%) year 1, and 14 CKD and 31 CKD-T patients (94%) year 3. The echocardiographic examinations were carried out using a standard system (Vivid 7, GE VingMed Ultrasound, version 108.0.1, Horten, Norway). A two-dimensional guided M-mode measurement, conventional pulse wave Doppler (PWD), and color-coded tissue Doppler imaging (cc-TDI) were performed according to the American Society of Echocardiography (ASE) guidelines [31, 32]. Left ventricular mass index (LVMI) was assessed (left ventricular mass/height^2.7^) [31, 33] as well as the presence of LVH [34]. Left ventricular diastolic function was evaluated with cc-TDI analyzing the peak myocardial velocities (cm/sec) during early (e´) and late (a´) diastole. The mean velocities of the septal and lateral margins of the mitral annulus were assessed for the e´/a´ ratio, according to recommendations [32]. The diastolic function was also assessed by PWD measuring mitral inflow velocity in early (E) diastole, and the PWD E/cc-TDI e´ ratio was calculated [35]. We used both raw data and calculated z-scores for cc-TDI é and PWD E analyses with cut-offs to define left ventricular diastolic dysfunction set at < 5th percentile [36]. An ejection fraction (EF) < 50% was used to define left ventricular systolic dysfunction. The detailed methods, longitudinal changes, and intra-observer variability of these echocardiographic analyses have recently been published [13].

### Statistical analysis

Statistical analyses were performed using Stata (StataCorp, TX, USA, version 16.0). Results are expressed as mean ± standard deviation and median [range]. Univariate analyses at baseline were performed using *t*-test or Wilcoxon rank-sum test for comparisons between groups.

For univariate and multivariate longitudinal analyzes, linear mixed models with a restricted maximum likelihood (reml) approach were used, which includes a random subject effect taking into account that a subject is measured several times. Non-normally distributed outcome variables were log-transformed before analyses.

The primary outcomes in the linear mixed models were log NT-proBNP, log GFR-adjusted NT-proBNP, log hs-cTnT, log GFR-adjusted hs-cTnT, and log hs-cTnI. The models included both baseline and follow-up measurements for the independent variables. In a sub-analysis, two different methods (Architect and DxI) for analyzing hs-cTnI were also assessed. Secondary outcomes were markers of cardiovascular morbidity; LVMI, LVH, cc-TDI e´ z-score, and PWD E z-score, as well as cc-TDI e´/a´ and PWD E/cc-TDI e´. Variables with *p*-values < 0.10 in univariate models were tested in the multivariable models to fit the best model. Due to potential confounding, patient group (CKD or CKD-T), age at baseline (years), and GFR (ml/min/1.73 m^2^) were forced into the model. A *p*-value < 0.05 was considered statistically significant.

**Fig. 1:**
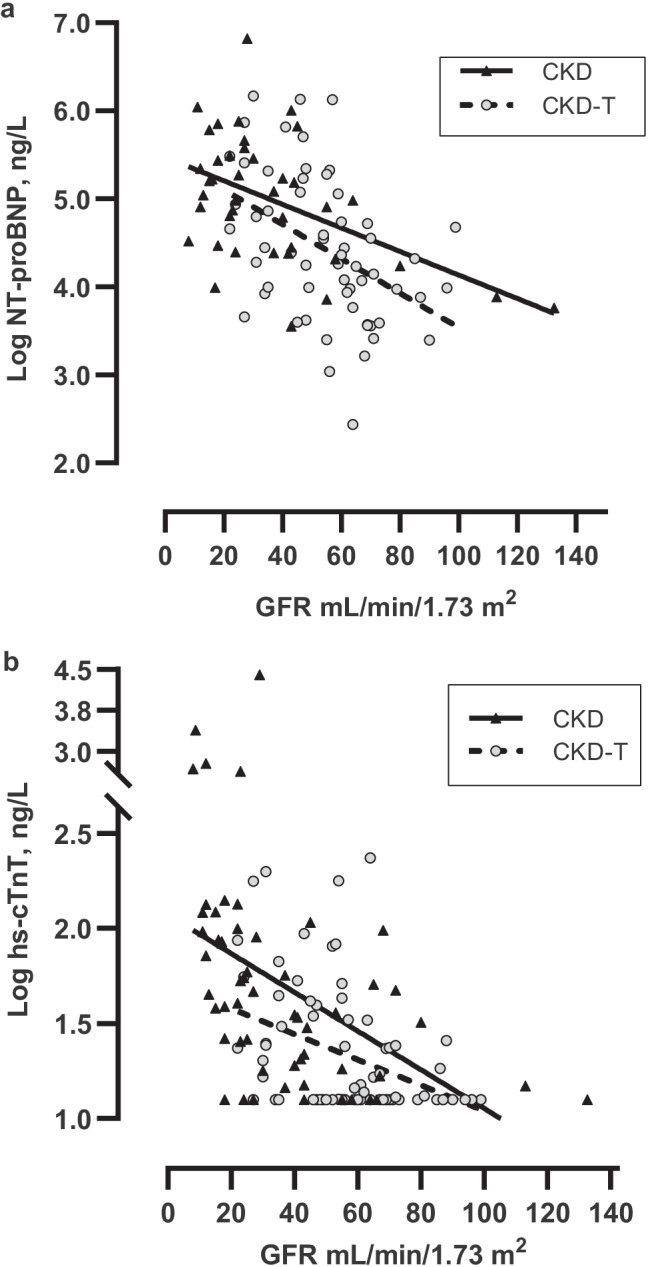
**a**–**b** Longitudinal levels of log NT-proBNP and log hs-cTnT versus GFR were analyzed using a linear regression with a cluster-robust variance–covariance matrix. Data are presented graphically for CKD patients and CKD-T patients separately. Both log NT-proBNP and log hs-cTnT were inversely associated with GFR in a linear fashion in both CKD (*β* =  − 0.01, *p* < 0.001 and *β* =  − 0.01, *p* = 0.001) and CKD-T patients (*β* =  − 0.02, *p* = 0.004 and *β* =  − 0.007, *p* = 0.009)

Subjects initiating kidney replacement therapy (dialysis or kidney transplant) during the study were analyzed separately following transplantation, resulting in potential bias due to nonrandom missing values as subjects with the most advanced disease were “lost to follow-up.” These patients were assessed to estimate the immediate effect of transplantation using a Mann–Whitney *U* test*.*

**Fig. 2:**
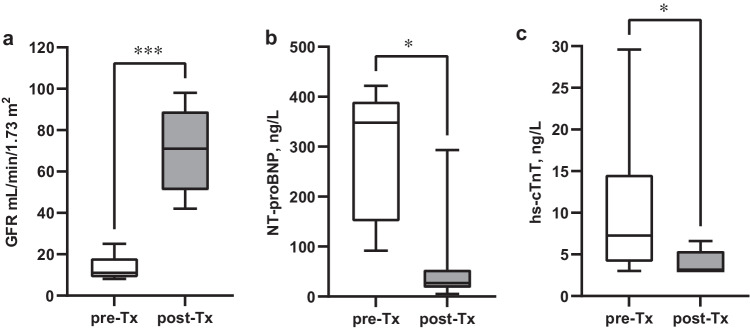
**a**–**c** Changes in NT-proBNP, hs-cTnT, and GFR levels in the 7 CKD patients who were transplanted during follow-up. Differences tested before and after kidney transplantation using Mann–Whitney *U* test; NT-proBNP *p < 0.05, hs-cTnT *p < 0.05, and GFR ***p < 0.001. The median post-transplant time was 1.1 years (range 0.5–1.1 years)

**Fig. 3:**
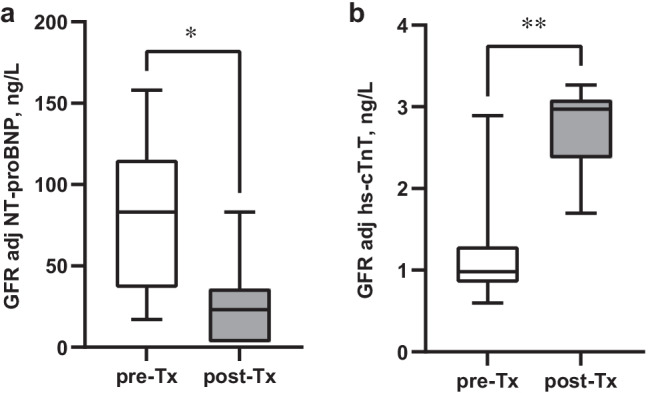
**a–b** Changes in GFR-adjusted NT-proBNP and GFR-adjusted hs-cTnT levels in the 7 CKD patients who were transplanted during follow-up. Differences tested before and after kidney transplantation using Mann–Whitney *U* test; GFR-adjusted NT-proBNP *p < 0.05, GFR-adjusted hs-cTnT **p < 0.01. The median post-transplant time was 1.1 years (range 0.5–1.1 years)

## Results

### Clinical characteristics at baseline

The baseline clinical characteristics of 22 CKD and 26 CKD-T patients are summarized in Table 1. There was no significant difference in mean time of follow-up in CKD (3.3 ± 0.4 years) and CKD-T patients (3.2 ± 0.6 years); *p* = 0.42. There were no dropouts during the study among CKD-T patients, but in total, 7 (32%) CKD patients were transplanted during the follow-up period. Data obtained after transplantation in this subgroup of patients were analyzed separately.

**Table 1:** Clinical characteristics at baseline

Baseline characteristics	CKD	CKD-T	*p*-value
	*n* = 22	*n* = 26	
Age, year	8.1 [0.8–14.5]	11.9 [3.3–16.8]	0.08
Duration of CKD, year	4.5 [0.8–14.5]	2.5 [0.4–13.2]	** < 0.001**
Time after transplantation, year		4.6 [0.9–14.0]	
BMI, z-score	− 0.09 ± 0.22	0.94 ± 0.22	**0.002**
- Obesity, *n* (%)	1 (4.5)	6 (23)	0.07
GFR, mL/min/1.73 m^2^	25.5 [8.8–68]	58 [30–99]	** < 0.001**
- GFR ≥ 60 mL/min/1.73 m^2^, *n* (%)	4 (18.2)	13 (50)	
- GFR 15–59 mL/min/1.73 m^2^, *n* (%)	14 (63.6)	13 (50)	
- GFR < 15 mL/min/1.73 m^2^, *n* (%)	4 (18.2)	0	**0.01**
Systolic blood pressure, z-score	0.58 ± 1.3	0.60 ± 0.98	0.96
Diastolic blood pressure, z-score	0.55 ± 0.90	0.33 ± 0.85	0.40
- Hypertension (BP ≥ 95th percentile and/or antihypertensives)	12 (55)	15 (58)	0.83
Albuminuria, *n* (%)	11 (50)	8 (31)	0.18
Medications:			
- Immunosuppressants, *n* (%)	1 (4.5)	26 (100)	** < 0.001**
- Antihypertensives, *n* (%)	11 (50)	13 (50)	1.0

*CKD* chronic kidney disease, *CKD-T* chronic kidney disease with kidney transplant, *BMI* body mass index, *GFR* glomerular filtration rate, *BP* blood pressure

### Cardiovascular risk markers and cardiac status at baseline

Cardiovascular risk was assessed according to the presence of anemia, inflammation, and deranged calcium–phosphorus balance as presented in Table 2. As expected, anemia and hyperparathyroidism were common in both groups, while low-grade inflammation and elevated calcium–phosphorus levels were less prevalent. Cardiac biomarkers and echocardiographic findings are presented in Table 3. The prevalence of high NT-pro-BNP was 27% in CKD and 11% in CKD-T patients, and similarly elevated hs-cTnT was present in 32% of CKD and 8% of CKD-T patients. Both NT-proBNP (median 155.1 vs. 78.0 ng/L; *p* = 0.02) and hs-cTnT (median 5.0 vs. 3.0; *p* = 0.02) were higher in CKD compared with CKD-T patients. In contrast, while GFR adjusted levels were generally lower in both groups, GFR-adjusted hs-cTnT was instead higher among CKD-T patients than for CKD patients (median 2.5 vs. 1.6 ng/L; *p* = 0.001), while GFR-adjusted NT-proBNP levels were similar in the two patient groups (*p* = 0.47). The levels for hs-cTnI (Architect and DxI) were lower than for hs-cTnT, and no patient had hs-cTnI (Architect or DxI) levels above the upper reference limit.

**Table 2:** Laboratory data at baseline

Laboratory data	CKD	CKD-T	p-value
Hemoglobin, g/L	116.7 ± 11.6	119.5 ± 10.3	0.37
Anemia (< 5th percentile), *n* (%)	9/22 (41%)	8/26 (31%)	0.46
hs-CRP, mg/L	0.42 [0.16–6.0]	0.29 [0.16–19.7]	0.78
Low-grade inflammation (> 3 mg/L), *n* (%)	2/22 (9%)	3/26 (11.5%)	0.78
i-PTH, ng/L	128 [20–391]	75.5 [35–126]	**0.01**
Hyperparathyroidism (> 97.5th percentile), *n* (%)	16/21 (76%)	13/24 (54%)	0.12
Albumin adjusted Calcium, mmol/L	2.4 ± 0.11	2.4 ± 0.10	0.84
Hypercalcemia (> 97.5th percentile), *n* (%)	2/21 (9.5%)	1/26 (4%)	0.42
Phosphate, mmol/L	1.5 ± 0.27	1.4 ± 0.24	0.07
Hyperphosphatemia (> 97.5th percentile), *n* (%)	2/22 (9%)	1/25 (4%)	0.24

**Table 3:** Cardiac markers and echocardiographic data at baseline

Cardiac data	CKD	CKD-T	*p*-value
LVMI, g/m^2.7^	34.8 [23.0–53.4]	36.9 [23.9–74.8]	0.24
LVH, n (%)	4/19 (21%)	5/18 (28%)	0.63
Cc-TDI e´/a´	3.1 [2.1–6.3]	2.4 [1.6–4.4]	0.21
Cc-TDI lat e´, z-score	− 0.33 ± 0.81	-0.82 ± 0.84	0.09
Cc-TDI lat e´ z-score < 5th percentile, *n* (%)	1/17 (6%)	3/18 (17%)	0.32
PWD E/A	1.8 [1.2–2.5]	1.6 [1.1–2.4]	0.43
PWD E, z-score	− 0.23 ± 0.62	0.01 ± 1.1	0.43
PWD E z-score < 5th percentile, *n* (%)	1/19 (5%)	0/18	0.32
PWD E/TDI e´	9.0 [7.0–11.9]	10.4 [7.0–17.5]	0.28
EF, %	67 [62–82]	71.5 [62–79]	0.34
EF < 50%, *n* (%)	0/15	0/18	
NT-proBNP, ng/L	155.1 [54.1–288.1]	78.0 [11.5–477]	**0.02**
NT-proBNP > 97.5th percentile, *n* (%)	4/15 (27%)	2/18 (11%)	0.25
GFR adjusted NT-proBNP	49 [12–109]	51 [9–193]	0.47
GFR adj NT-proBNP > 97.5th percentile, *n* (%)	0/15	1/18 (6%)	0.35
Hs-cTnT, ng/L	5.0 [3.0–29.6]	3.0 [3.0–9.5]	**0.02**
Hs-cTnT > 97.5th percentile,* n* (%)	7/22 (32%)	2/25 (8%)	**0.04**
GFR adjusted hs-cTnT	1.6 [0.75–5.5]	2.5 [1.1–5.7]	**0.001**
GFR adj hs-cTnT > 97.5th percentile, *n* (%)	0/22	0/26	
Hs-cTnI, ng/L (DxI)	0.9 [0.0–7.1]	1.1 [0.1–2.3]	0.74
Hs-cTnI, ng/L (DxI) > 99th percentile, *n* (%)	0/22	0/26	
Hs-cTnI, ng/L (Architect)	1.1 [0.0–11.0]	0.6 [0.0–2.1]	0.14
Hs-cTnI, ng/L (Architect) > 95th percentile, *n* (%)	0/19	0/24	

*LVMI* left ventricular mass index, *LVH* left ventricular hypertrophy, *Cc-TDI* color-coded tissue Doppler imaging, *PWD* pulse wave Doppler, *EF* ejection fraction, *NT-proBNP* N-terminal pro-B-type natriuretic peptide, *Hs-cTnT* high-sensitive cardiac-specific troponin T, *Hs-cTnI* high-sensitive cardiac-specific troponin I

Cardiac data at baseline show a prevalence of LVH of 21% in CKD and 28% in CKD-T patients (Table 3). While no patient presented with left ventricular systolic dysfunction (EF < 50%), diastolic dysfunction defined as TDI e´ z-score < 5th percentile was present in 6% of CKD and 17% of CKD-T patients.

### Longitudinal patterns

In univariate analyses of changes during follow-up, mean GFR was stable over time in the CKD group (*β* = 1.01, *p* = 0.30), while mean GFR declined with an annual rate of 2.4 mL/min/1.73 m^2^ in CKD-T patients (*p* = 0.002). Systolic and diastolic blood pressure z-scores as well as BMI z-score, inflammatory profile, and hemoglobin levels remained unchanged during the 3-year follow-up period in all study participants (data not shown). Calcium–phosphorus balance changed significantly in both groups, where phosphate (*β* =  − 0.07, *p* = 0.005) and i-PTH (*β* =  − 11.8, *p* = 0.05) decreased in CKD patients and albumin-adjusted calcium (*β* =  − 0.01, *p* = 0.01) and phosphate (*β* =  − 0.05, *p* = 0.002) decreased in CKD-T patients over time.

Only log hs-cTnI (Architect, *β* = 0.15, *p* = 0.005), but none of the other cardiac biomarkers, changed significantly during the follow-up period in CKD patients. There was no significant change in cardiac biomarkers in the CKD-T group (data not shown). Regarding cardiac status, none of the measured echocardiographic markers changed over time in CKD-T patients and only PWD E/cc-TDI é decreased significantly in the CKD group; *β* =  − 0.17, *p* = 0.05.

### Association between kidney function and cardiac biomarkers

Multivariate analyses of follow-up data reveal a strong association between kidney function and both log NT-proBNP (*β* =  − 0.01, *p* = 0.01) and log hs-cTnT (*β* =  − 0.007, *p* = 0.01) (Table 4). To visualize this correlation, these cardiac markers were plotted graphically in relation to kidney function in both patient groups (Fig. 1a–b). Both log NT-proBNP and log hs-cTnT were inversely associated with GFR in a linear fashion in both CKD (*β* =  − 0.01, *p* < 0.001 and *β* =  − 0.01, *p* = 0.001) and CKD-T patients (*β* =  − 0.02, *p* = 0.004 and *β* =  − 0.007, *p* = 0.009). In contrast, log hs-cTnI was not associated with GFR (*p* = 0.78 for DxI and *p* = 0.18 for Architect).

**Table 4:** Multivariate correlations of cardiac biomarkers using longitudinal data

Outcome	Variable	*β*	95% CI	*p*-value	Model-p	*R*^2^
Log NT-proBNP	CKD versus CKD-T	− 0.24	(− 0.69, 0.22)	0.30	< 0.001	0.38
	Age at baseline, years	− 0.004	(− 0.07, 0.07)	0.90		
	Time with disease, years	− 0.03	(− 0.09, 0.04)	0.40		
	Hemoglobin, g/L	− 0.01	(− 0.03, 0.001)	0.08		
	GFR, mL/min/1.73 m^2^	− 0.01	(− 0.02, − 0.002)	**0.01**		
	Systolic BP, z-score	0.09	(− 0.05, 0.23)	0.20		
	Antihypertensives, 0/1	0.31	(− 0.02, 0.63)	0.06		
	LVMI, g/m^2.7^	0.02	(0.0001, 0.04)	**0.05**		
Log hs-cTnT	CKD versus CKD-T	− 0.12	(− 0.35, 0.12)	0.34	< 0.001	0.36
	Age at baseline, years	− 0.001	(− 0.03, 0.03)	0.92		
	Sex, 0/1	0.25	(0.02, 0.47)	**0.03**		
	BMI, z-score	− 0.08	(− 0.17, 0.004)	0.06		
	GFR, mL/min/1.73 m^2^	− 0.007	(− 0.01, − 0.002)	**0.01**		
	Albuminuria, 0/1	0.14	(− 0.03, 0.31)	0.11		
	cc-TDI e´/a´	− 0.09	(− 0.17, − 0.001)	**0.05**		
Log hs-cTnI (Architect)	CKD versus CKD-T	0.07	(− 0.42, 0.55)	0.78	0.01	0.11
	Age at baseline, years	− 0.06	(− 0.12, − 0.005)	**0.03**		
	Hemoglobin, g/L	0.02	(0.007, 0.04)	**0.005**		
	GFR, mL/min/1.73 m^2^	− 0.007	(− 0.02, 0.003)	0.18		
Log hs-cTnI (DxI)	CKD versus CKD-T	0.34	(− 0.17, 0.85)	0.19	0.004	0.11
	Age at baseline, years	− 0.06	(− 0.13, 0.005)	0.07		
	Sex, 0/1	0.38	(− 0.10, 0.87)	0.12		
	GFR, mL/min/1.73 m^2^	− 0.001	(− 0.01, 0.008)	0.78		
	Albuminuria, 0/1	0.36	(0.03, 0.69)	**0.04**		
	LVMI, g/m^2.7^	0.01	(− 0.005, 0.03)	0.15		
	cc-TDI e´/a´	− 0.13	(− 0.29, 0.04)	0.13		

*CKD* chronic kidney disease, *CKD-T* chronic kidney disease with kidney transplant, *BMI* body mass index, *GFR* glomerular filtration rate, *BP* blood pressure, *LVMI* left ventricular mass index, *Cc-TDI* color-coded tissue Doppler imaging, *NT-proBNP* N-terminal pro-B-type natriuretic peptide, *Hs-cTnT* high-sensitive cardiac-specific troponin T, *Hs-cTnI* high-sensitive cardiac-specific troponin I

Cardiac biomarkers and GFR were also assessed in the 7 CKD patients who were transplanted during follow-up, comparing levels before and after transplantation (Fig. 2a–c). This analysis reveals a marked decline in hs-cTnT (*p* = 0.02) and NT-proBNP levels (*p* = 0.01) following kidney transplantation, corresponding to the rise in GFR (*p* < 0.001). While this trend was unchanged for GFR-adjusted NT-proBNP that decreased after transplantation (*p* = 0.03), GFR-adjusted hs-cTnT increased instead (*p* = 0.002), (Fig. 3a–b). There was no significant change in log hs-cTnI (Dxl or Architect) before and after kidney transplantation (*p* = 0.24 and *p* = 0.47).

### Longitudinal associations between cardiac biomarkers and echocardiographic data

**Fig. 4:**
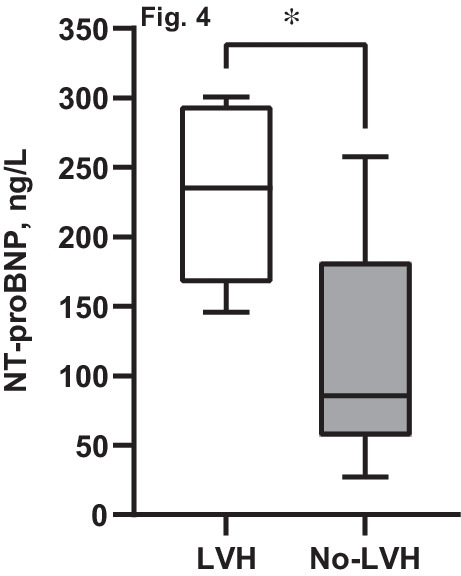
Differences in NT-proBNP for CKD and CKD-T patients with or without LVH. Analyses made using Mann–Whitney *U* test; *p < 0.05

Multivariate analyses of longitudinal associations for cardiac biomarkers show that elevated log NT-proBNP was associated with increased LVMI (*β* = 0.02, *p* = 0.05) (Table 4). Indeed, patients with LVH at baseline had higher NT-proBNP (235 [146–301] ng/L) compared with patients without LVH (86 [11–477] ng/L), *p* = 0.02, (Fig. 4). To further adjust for the strong association between kidney function and NT-proBNP presented above, GFR-adjusted NT-proBNP [29] was also assessed as an outcome measure in a sub-analysis. GFR-adjusted NT-proBNP was strongly associated with LVMI in univariate analyses; *β* = 0.02, *p* = 0.01, and this significant correlation remained after adjustments in a multivariate model (*β* = 0.02, *p* = 0.02).

High log hs-cTnT over-time was associated with an affected LV diastolic function assessed as low cc-TDI e´/a´ (*β* =  − 0.09, *p* = 0.05). As for NT-proBNP, we also assessed correlations for GFR-adjusted levels of log hs-cTnT. In this multivariate model, the significance for TDI e´/a´ noted previously disappeared (*β* =  − 0.05, *p* = 0.26). As for log hs-cTnI (Dxl), a significant association was seen for both high LVMI (*β* = 0.02, *p* = 0.03) and low cc-TDI e´/a´ (*β* =  − 0.19, *p* = 0.03) in univariate analyses, but this did not remain after adjustments in the multivariate model (Table 4). Hs-cTnI (Architect) was not associated with any cardiac measure.

## Discussion

The burden of CVD in CKD is high, and the incentives to find stable cardiac biomarkers that may forecast future CVD are strong. Both cTn’s and NT-proBNP are associated with increased risk of heart failure and cardiovascular death in several populations [6, 37]. It is also evident that these biomarkers are associated with early subclinical markers of CVD in CKD patients [38]. Identifying biomarkers that can improve diagnostic accuracy reflecting early cardiovascular changes like LV remodeling and dysfunction could improve outcome for these patients [15]. However, longitudinal data in CKD are limited, especially regarding pediatric studies.

The present study analyzes associations between longitudinal levels of high-sensitive cardiac-specific troponin T (hs-cTnT), troponin I (hs-cTnI), and NT-proBNP and early subclinical pathological changes in cardiac structure and function in a cohort of pediatric CKD and CKD-T patients. Echocardiography was used to analyze the presence of increased LVMI and affected LV diastolic function as early markers of cardiac disease.

The results of this study suggest that levels of NT-proBNP and hs-cTnT are elevated in pediatric CKD and CKD-T patients and support previous studies revealing a strong correlation with GFR [19, 39]. Indeed, elevated levels might not only reflect increased cardiac release, but also decreased urinary clearance which complicates interpretation of these biomarkers in CKD. It has therefore been suggested that adjusting levels of cardiac biomarkers to kidney function may give a better prognostic value [29, 30, 37]. Using an algorithm adjusting NT-proBNP and hs-cTnT for GFR based on adult data [29, 30], both GFR-adjusted levels as well as unadjusted data were analyzed in this study. Following multivariate adjustments, both GFR-adjusted NT-proBNP and NT-proBNP were associated with elevated LVMI, indicating its important role in early cardiac abnormalities already in pediatric patients. Similarly, patients with LVH at baseline had higher levels of NT-proBNP compared with patients without LVH. These findings are in line with other pediatric and adult studies revealing a strong association between NT-proBNP, LVMI, and LVH [17, 19, 40].

Hs-cTnT was in this study not correlated to LVMI but with a decreased LV diastolic function revealed as low TDI e´/a´. Similar findings have been reported in large-scale cross-sectional and longitudinal studies of adult healthy populations as well as CKD patients [15, 41], but few pediatric studies have previously presented data on correlations between cardiac-specific troponins and cardiac outcome. Pediatric studies of children with CKD grade 3–5 have shown that cTnT was associated with LVH and systolic dysfunction [16], as well as low LV contractility [18]. As GFR-adjusted hs-cTnT was not associated with any cardiac measure in our study, it is at this stage difficult to state the true relevance of the association between hs-cTnT and LV diastolic function in pediatric CKD. In addition, GFR-adjusted hs-cTnT was higher among CKD-T patients compared with CKD patients, while the opposite relationship was seen for unadjusted hs-cTnT. This could be explained by the strong impact of GFR, but the GFR adjusting equation that was used could also be one causal factor. Indeed, as the algorithm used is derived from clinical studies conducted on adult patients [30], it is not yet proven to be reproducible in the pediatric age. In addition, a similar trend was not seen for NT-proBNP where another GFR adjusting equation was used [29]. Another important aspect is that GFR-adjusted hs-cTnT increased in those CKD patients who were transplanted during the study, which indicates that parameters related to the transplantation itself could be of importance. Indeed, the cardiotoxic drugs used in CKD-T patients have been shown to affect these cardiac markers differently [42]. It is thus reasonable to assume that hs-cTnT in CKD and CKD-T patients is influenced by a complex interplay between both cardiac and renal status as well as different treatments.

In line with previous pediatric studies and in contrast to the other cardiac biomarkers, hs-cTnI was not associated with kidney function [43]. Similarly, the levels of hs-cTnI were not as high as NT-ProBNP and hs-cTnT. The different importance of hs-cTnT and hs-cTnI in this patient group has been presented previously [18]. Still, controversy exists as some studies show that cTnT but not cTnI is an important indicator of cardiac disease and death [18, 44], while other reports show that cTnI is indeed a strong predictor for adverse cardiovascular events in patients with CKD [45].

That findings vary for the three cardiac biomarkers analyzed in this study could be explained by the limited number of patients analyzed, which affect the possibility to reach power and statistical significance in all analyses. Another reason could be explained by their different physiological actions. For example, cardiac-specific troponins are peptides released from cardiac muscle cells activated during muscle contraction, while BNP is a hormone secreted by cardiomyocytes in response to stretching of the ventricles due to increased intravascular volume. Increased troponin levels are detected in cases with cardiomyocyte damage like myocardial ischemia and necrosis in acute coronary syndromes [2, 46], and NT-proBNP is considered a classical biomarker of heart failure [3, 14]. Several large-scale studies have demonstrated associations between elevations of hs-cTnT and NT-proBNP with increased risk of CV and all-cause deaths in the general population [47–49]. These biomarkers are also associated with structural and functional cardiac abnormalities in CKD patients [14, 15, 40], but longitudinal data are limited [6] especially in the pediatric population [16, 18]. From the results in the present study, we conclude that NT-proBNP, hs-cTnT, and hs-cTnI could give complementary biochemical information in pediatric CKD and that they cannot replace each other.

There are some important limitations to this study. First, while we had only 3% missing data for hs-cTnT and hs-cTnI, we had 25% missing values for NT-proBNP over follow-up. We also had 13% missing values for the echocardiographic measurements, which might have affected the results on the outcome analysis. Finally, we have important bias in the CKD group as seven (32%) patients were transplanted during follow-up, being those with the worst kidney function who dropped out.

In summary, we report that NT-proBNP and hs-cTnT are strongly associated with GFR with increasing levels as kidney function deteriorates. Both cardiac biomarkers are closely related to abnormalities of left ventricular structure (increased LVMI) or function (decreased TDI e´/a´) in pediatric CKD and CKD-T patients, but the correlation only remains for NT-proBNP after adjustments for GFR. Importantly, it is possible that these cardiac biomarkers are more markers of disease than causally linked to CVD in these patients. Therefore, future studies need to assess whether and which GFR-adjusted values should be used when interpreting cardiac biomarkers in pediatric nephrology research. Further, it is important to increase knowledge of the underlying biological mechanisms and to explore whether targeting these biomarkers with therapy might reduce the risk of future CVD.

## Supplementary Information

Below is the link to the electronic supplementary material.

Graphical Abstract 5481 (PPTX 284 KB)

## Data Availability

The datasets generated during and/or analyzed during the current study are not publicly available due to ongoing studies including a long-term follow-up of this cohort.
