# Supplementary material for: Cardiac biomarkers in pediatric CKD—a prospective follow-up study
Source: Pediatr Nephrol. 2022 Mar 16;37(12):3165–75. doi: 10.1007/s00467-022-05481-w (PMC9587089; doi:10.1007/s00467-022-05481-w)
Supplement: Supplementary file 1 — Graphical Abstract 5481 (PPTX 284 KB) [file 467_2022_5481_MOESM1_ESM.pptx]

## Slide 1
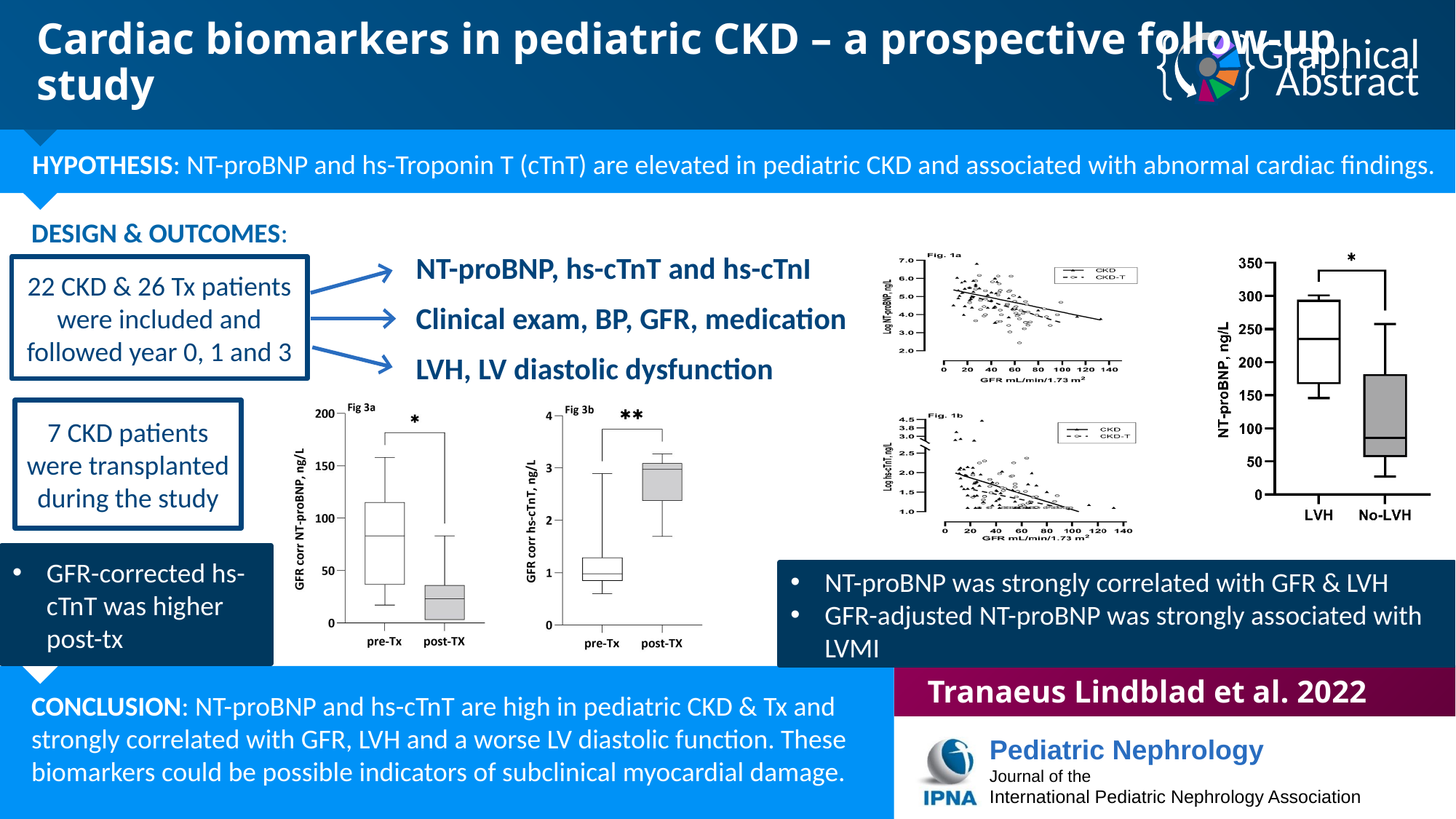

Cardiac biomarkers in pediatric CKD – a prospective follow-up
study
HYPOTHESIS: NT-proBNP and hs-Troponin T (cTnT) are elevated in pediatric CKD and associated with abnormal cardiac findings.
DESIGN & OUTCOMES:
NT-proBNP, hs-cTnT and hs-cTnI
22 CKD & 26 Tx patients were included and
followed year 0, 1 and 3
Clinical exam, BP, GFR, medication
LVH, LV diastolic dysfunction
7 CKD patients were transplanted during the study
GFR-corrected hs-cTnT was higher post-tx
NT-proBNP was strongly correlated with GFR & LVH
GFR-adjusted NT-proBNP was strongly associated with LVMI
Tranaeus Lindblad et al. 2022
CONCLUSION: NT-proBNP and hs-cTnT are high in pediatric CKD & Tx and strongly correlated with GFR, LVH and a worse LV diastolic function. These biomarkers could be possible indicators of subclinical myocardial damage.
